# Do not try this at home: patterns and outcomes of radiation dose de-escalation in p16-positive oropharyngeal carcinoma

**DOI:** 10.3389/fonc.2026.1929183

**Published:** 2026-09-07

**Authors:** Asher J. Shin, Ethan D. Shin, Victor Lee, Edward Christopher Dee, Sean McBride, Yao Yu, Nadeem Riaz, Jung Julie Kang

**Affiliations:** 1 Department of Radiation Oncology, Memorial Sloan Kettering Cancer Center, New York, NY, United States; 2 Department of Therapeutic Radiology, Yale University School of Medicine, New Haven, CT, United States

**Keywords:** head and neck cancer, HPV-associated oropharyngeal cancer, p16 positive, radiation therapy, radiation de-escalation

## Abstract

**Introduction:**

Radiation dose de-escalation for human papillomavirus (HPV)-associated oropharyngeal squamous cell carcinoma (OPSCC) has been actively investigated to reduce long-term toxicity. Following the recent failure of randomized non-inferiority trials, the real-world adoption and outcomes of de-escalated radiation remain unclear. We evaluated contemporary patterns of dose de-escalation and associated overall survival (OS) in a national cohort.

**Methods:**

The National Cancer Database was queried for patients diagnosed from 2018–2022 with p16-positive OPSCC meeting NRG-HN005 eligibility criteria (cT1–2N1 or cT3N0–1) treated with definitive external beam radiation therapy without primary surgery. Dose-de-escalated radiation therapy (DDRT) was defined as 50.0–65.9 Gy; standard-dose radiation therapy (SDRT) as ≥66.0 Gy. Multivariable logistic regression identified predictors of DDRT. OS was estimated using Kaplan–Meier methods with log-rank testing and 2-month landmark analysis.

**Results:**

Among 14,869 patients, 1,119 (7.5%) received DDRT. DDRT was more frequently delivered at academic centers (50.3% vs 40.0%, p<0.001). On multivariable analysis, academic facility type (Odds Ratio: 1.76, 95% Confidence Interval 1.31-2.43, p<0.001), more recent year of diagnosis, and geographic region were independently associated with DDRT. Three-year OS was inferior with DDRT compared with SDRT (82.1% vs 88.9%, p<0.001). Among patients receiving first-course chemotherapy, DDRT remained associated with worse OS (82.5% vs. 89.8%, p<0.001). No significant difference was observed among patients not receiving chemotherapy; however, this subgroup was small and likely underpowered to detect a meaningful difference. Landmark analysis yielded consistent findings.

**Conclusion:**

Radiation dose de-escalation remains infrequent in contemporary U.S. practice but is more commonly delivered at academic centers. In this population-based analysis, de-escalation was associated with inferior overall survival, aligning with recent randomized evidence. Reduced-dose radiation should remain investigational pending biomarker-guided selection strategies.

## Introduction

Human papillomavirus (HPV)-associated, p16-positive oropharyngeal squamous cell carcinoma (OPSCC) represents a distinct clinical entity characterized by favorable prognosis and excellent response to standard chemoradiation. Patients with p16-positive disease are often younger, with fewer comorbidities, and demonstrate high rates of locoregional control and overall survival (OS) compared with HPV-negative OPSCC (1–3). As a result, there has been growing interest in treatment de-intensification strategies, particularly radiation dose de-escalation, to reduce long-term toxicities such as dysphagia, xerostomia, and feeding tube dependence, which significantly impact quality of life in survivors (4–15).

Early phase studies suggested that select low-risk p16-positive patients could safely receive lower radiation doses without compromising disease control, fueling enthusiasm for prospective de-escalation trials (8–13). NRG-HN005 randomized eligible patients to 70 Gy over 6 weeks with cisplatin, 60 Gy over 6 weeks with cisplatin, or 60 Gy over 5 weeks with nivolumab. Two-year progression-free survival was 98.1%, 88.6%, and 90.3%, respectively, and neither experimental arm met the phase II non-inferiority criterion (15). The study included patients with stage T1-2N1M0 or T3N0-N1M0 disease and limited smoking history (≤10 pack-years). While initial data demonstrated the safety and feasibility of dose reduction in highly selected patients, the trial ultimately failed to meet non-inferiority criteria for progression-free survival (PFS) in the experimental arms, in part due to the exceptionally favorable outcomes in the standard therapy arm (15). These findings underscore the difficulty of safely reducing radiation intensity in p16-positive OPSCC outside of carefully controlled trial populations.

Despite these results, the degree to which dose de-escalation has been adopted in real-world clinical practice remains largely unexplored. Understanding contemporary patterns of care is critical, particularly because academic centers may be more likely to implement emerging strategies based on early-phase evidence or trial participation, whereas community centers may adhere more strictly to established guidelines (16, 17). Large national datasets such as the National Cancer Database (NCDB) allow for the examination of treatment patterns across diverse practice settings and patient populations, providing insight into the translation of clinical trial findings into everyday oncology practice (18–20).

In this study, we aimed to evaluate patterns of radiation dose de-escalation in p16-positive OPSCC patients treated with primary chemoradiation, excluding those who underwent surgery. We specifically examined differences in practice between academic and non-academic centers, with the goal of characterizing contemporary adoption of dose reduction strategies and identifying potential institutional disparities in the management of this favorable-risk population.

## Materials and methods

### Data source and study population

We queried NCDB for patients diagnosed with p16-positive OPSCC between 2018 and 2022 (21). The NCDB is a hospital-based registry capturing approximately 70% of newly diagnosed cancers in the United States, including detailed patient demographics, tumor characteristics, treatment modalities, and facility type. Patients were included if they had p16-positive status, AJCC 8^th^ edition clinical stage T1-2N1 or T3N0–1 per HN005 criteria, and were treated with definitive radiation therapy with or without first-course chemotherapy. Patients who underwent primary surgical resection, including transoral robotic surgery or open procedures, were excluded to isolate the effects of radiation therapy.

### Radiation dose classification

Radiation dose was categorized into two groups for analysis. Dose de-escalation was defined as a total radiation dose of 5000–6599 cGy, whereas standard therapy was defined as ≥6600 cGy. The NCDB provides phase dose and fraction counts but does not reliably encode fractions delivered per week so acceleration was therefore not incorporated. These cutoffs were selected based on prior publications investigating de-escalated radiation regimens in p16-positive oropharyngeal cancer.

### Covariates

Patient demographics included age at diagnosis, sex, race/ethnicity, insurance status, and year of diagnosis. Tumor characteristics included T stage, N stage, and AJCC 8th edition stage (22). Treatment facility type was classified as academic/research program versus community cancer program according to NCDB designations. Other covariates included comorbidity burden using the Charlson-Deyo Comobidity-Index.

### Statistical analysis

Descriptive statistics were used to summarize baseline patient and tumor characteristics, stratified by facility type and radiation dose group. Differences between groups were compared using chi-square tests for categorical variables and t-tests or Wilcoxon rank-sum tests for continuous variables, as appropriate. Descriptive analyses were performed using the tableone package in Python to generate summary tables (23). The primary outcome was the proportion of patients receiving dose de-escalated radiation, with a secondary focus on institutional differences between academic and non-academic centers. Multivariable logistic regression was used to identify factors associated with receiving de-escalated RT. OS was analyzed using a multivariable Cox proportional hazards regression model, including the same covariates used in the logistic regression analysis, with the addition of the DDRT variable. Given the large sample size and availability of clinically relevant covariates, we considered multivariable Cox regression an efficient and appropriate approach for confounding adjustment in this study. OS was also assessed using Kaplan–Meier estimates and compared with log-rank tests, with a 2-month landmark analysis conducted to account for early events. Multicollinearity among covariates was assessed using generalized variance inflation factors (GVIFs), with adjusted GVIFs (GVIF^(1/(2×Df))) used for interpretation. Patients diagnosed in 2022 were excluded from survival analysis because the most recent NCDB does not include survival data for recent patients due to limited follow-up. All analysis was performed with Python 3.7 and R 4.4.2. Models used complete cases without imputation.

## Results

**Figure 1:**
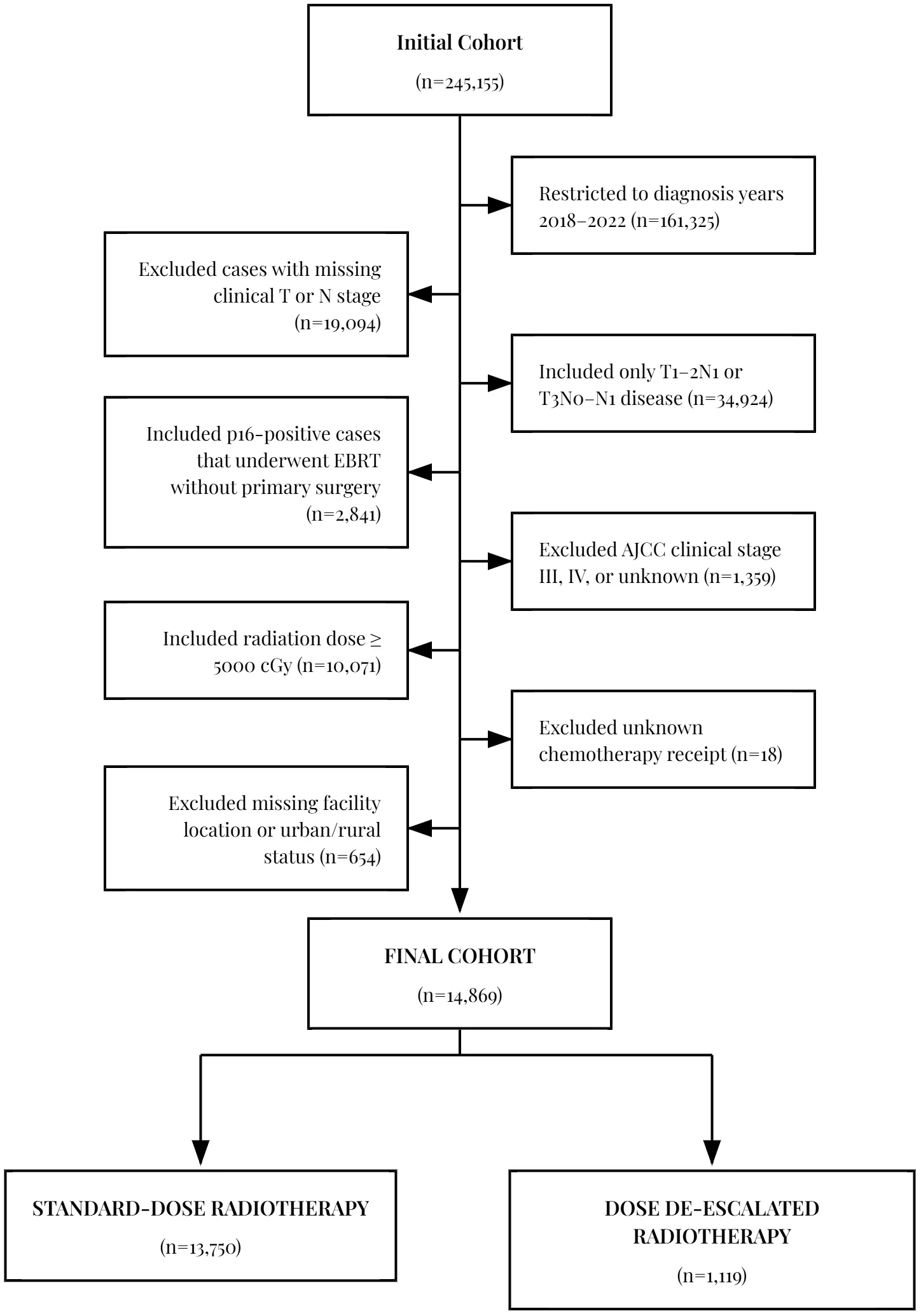
CONSORT-style cohort selection diagram. Flow diagram illustrating sequential inclusion criteria applied to the National Cancer Database to identify the final analytic cohort of patients with p16-positive oropharyngeal squamous cell carcinoma treated with definitive external beam radiation therapy.

A total of 14,869 patients with p16-positive oropharyngeal squamous cell carcinoma meeting NRG-HN005 eligibility criteria and treated with definitive external beam radiation therapy were identified between 2018 and 2022 (15) (**Figure 1**; **Table 1**). Patients in the DDRT cohort were slightly older, with a median age of 64 years (IQR 57–71) compared with 63 years (IQR 57–69) in the SDRT cohort (p<0.001). Of these, 1,119 patients (7.5%) received dose-de-escalated radiation therapy (DDRT; 50.0–65.9 Gy), while 13,750 patients (92.5%) received standard-dose radiation therapy (SDRT; ≥66.0 Gy).

**Table 1:** Patient, tumor, treatment, and facility characteristics stratified by radiation dose group.

		Grouped by dose de-escalation				
Variable	Category	Missing	Overall	SDRT	DDRT	P-value
n			14869	13750	1119	
Age, median [Q1,Q3]		0	63.0 [57.0,69.0]	63.0 [57.0,69.0]	64.0 [57.0,71.0]	<0.001
Sex, n (%)	Female	0	1706 (11.5)	1554 (11.3)	152 (13.6)	0.024
	Male		13163 (88.5)	12196 (88.7)	967 (86.4)	
Race, n (%)	Asian	0	135 (0.9)	126 (0.9)	9 (0.8)	0.626
	Black		694 (4.7)	650 (4.7)	44 (3.9)	
	Native American		36 (0.2)	34 (0.2)	2 (0.2)	
	Other		243 (1.6)	221 (1.6)	22 (2.0)	
	White		13761 (92.5)	12719 (92.5)	1042 (93.1)	
Year of Diagnosis, n (%)	2018	0	2372 (16.0)	2211 (16.1)	161 (14.4)	0.350
	2019		2951 (19.8)	2734 (19.9)	217 (19.4)	
	2020		2993 (20.1)	2775 (20.2)	218 (19.5)	
	2021		3341 (22.5)	3069 (22.3)	272 (24.3)	
	2022		3212 (21.6)	2961 (21.5)	251 (22.4)	
Facility Type, n (%)	Academic/Research Program (includes NCI-designated comprehensive cancer centers)	0	6057 (40.7)	5494 (40.0)	563 (50.3)	<0.001
	Community Cancer Program		974 (6.6)	910 (6.6)	64 (5.7)	
	Comprehensive Community Cancer Program		5044 (33.9)	4740 (34.5)	304 (27.2)	
	Integrated Network Cancer Program		2794 (18.8)	2606 (19.0)	188 (16.8)	
Facility Location, n (%)	East North Central (IL, IN, MI, OH, WI)	0	2839 (19.1)	2661 (19.4)	178 (15.9)	<0.001
	East South Central (AL, KY, MS, TN)		1016 (6.8)	926 (6.7)	90 (8.0)	
	Middle Atlantic (NJ, NY, PA)		1699 (11.4)	1579 (11.5)	120 (10.7)	
	Mountain (AZ, CO, ID, MT, NM, NV, UT, WY)		790 (5.3)	727 (5.3)	63 (5.6)	
	New England (CT, MA, ME, NH, RI, VT)		972 (6.5)	940 (6.8)	32 (2.9)	
	Pacific (AK, CA, HI, OR, WA)		1981 (13.3)	1834 (13.3)	147 (13.1)	
	South Atlantic (DC, DE, FL, GA, MD, NC, SC, VA, WV)		3296 (22.2)	2966 (21.6)	330 (29.5)	
	West North Central (IA, KS, MN, MO, ND, NE, SD)		1108 (7.5)	1014 (7.4)	94 (8.4)	
	West South Central (AR, LA, OK, TX)		1168 (7.9)	1103 (8.0)	65 (5.8)	
Urban/Rural Classification, n (%)	Metro: 250,000–999,999 population	0	3470 (23.3)	3218 (23.4)	252 (22.5)	0.384
	Metro: <250,000 population		1522 (10.2)	1417 (10.3)	105 (9.4)	
	Metro: ≥1 million population		7221 (48.6)	6679 (48.6)	542 (48.4)	
	Rural: <2,500 population, adjacent to metro		182 (1.2)	168 (1.2)	14 (1.3)	
	Rural: <2,500 population, not adjacent to metro		162 (1.1)	152 (1.1)	10 (0.9)	
	Urban: 2,500–19,999 population, adjacent to metro		887 (6.0)	808 (5.9)	79 (7.1)	
	Urban: 2,500–19,999 population, not adjacent to metro		430 (2.9)	392 (2.9)	38 (3.4)	
	Urban: ≥20,000 population, adjacent to metro		780 (5.2)	712 (5.2)	68 (6.1)	
	Urban: ≥20,000 population, not adjacent to metro		215 (1.4)	204 (1.5)	11 (1.0)	
Median Income Quartile in 2020, n (%)	$46,277 - $57,856	2335	2673 (21.3)	2483 (21.4)	190 (20.2)	0.435
	$57,857 - $74,062		3145 (25.1)	2920 (25.2)	225 (23.9)	
	$74,063+		5158 (41.2)	4762 (41.1)	396 (42.1)	
	< $46,277		1558 (12.4)	1429 (12.3)	129 (13.7)	
No High School Degree Quartile in 2020, n (%)	15.3%+	2302	1969 (15.7)	1826 (15.7)	143 (15.2)	0.386
	5.0% - 9.0%		4013 (31.9)	3729 (32.1)	284 (30.1)	
	9.1% - 15.2%		3362 (26.8)	3109 (26.7)	253 (26.9)	
	< 5.0%		3223 (25.6)	2961 (25.5)	262 (27.8)	
Insurance Status, n (%)	Insurance Status Unknown	0	135 (0.9)	125 (0.9)	10 (0.9)	0.017
	Medicaid		948 (6.4)	888 (6.5)	60 (5.4)	
	Medicare		5962 (40.1)	5458 (39.7)	504 (45.0)	
	Not Insured		284 (1.9)	260 (1.9)	24 (2.1)	
	Other Government		606 (4.1)	564 (4.1)	42 (3.8)	
	Private Insurance/Managed Care		6934 (46.6)	6455 (46.9)	479 (42.8)	
Radiation Location, n (%)	All RT at this facility	0	12559 (84.5)	11623 (84.5)	936 (83.6)	0.063
	All RT elsewhere		2133 (14.3)	1963 (14.3)	170 (15.2)	
	Boost radiation at this facility, regional elsewhere		10 (0.1)	9 (0.1)	1 (0.1)	
	Other/Unknown		144 (1.0)	137 (1.0)	7 (0.6)	
	Regional treatment at this facility, boost elsewhere		23 (0.2)	18 (0.1)	5 (0.4)	
Best Charlson Deyo Score, n (%)	0	0	11770 (79.2)	10897 (79.3)	873 (78.0)	0.190
	1		2021 (13.6)	1863 (13.5)	158 (14.1)	
	2		572 (3.8)	517 (3.8)	55 (4.9)	
	3 or more		506 (3.4)	473 (3.4)	33 (2.9)	
Clinical T Stage, n (%)	1	0	4129 (27.8)	3811 (27.7)	318 (28.4)	0.719
	2		7306 (49.1)	6753 (49.1)	553 (49.4)	
	3		3434 (23.1)	3186 (23.2)	248 (22.2)	
Clinical N Stage, n (%)	0	0	693 (4.7)	635 (4.6)	58 (5.2)	0.430
	1		14176 (95.3)	13115 (95.4)	1061 (94.8)	
Clinical Stage Group, n (%)	1	0	11182 (75.2)	10327 (75.1)	855 (76.4)	0.350
	2		3687 (24.8)	3423 (24.9)	264 (23.6)	
Chemotherapy Treatment Status, n (%)	Chemotherapy administered as first course therapy	0	323 (2.2)	296 (2.2)	27 (2.4)	<0.001
	Contraindicated due to patient risk factors		122 (0.8)	111 (0.8)	11 (1.0)	
	Multiagent chemotherapy administered as first course therapy		1797 (12.1)	1608 (11.7)	189 (16.9)	
	None		1945 (13.1)	1730 (12.6)	215 (19.2)	
	Not administered; patient/family refused		128 (0.9)	117 (0.9)	11 (1.0)	
	Not administered; physician recommended, but not given		7 (0.0)	7 (0.1)		
	Single-agent chemotherapy administered as first course therapy		10547 (70.9)	9881 (71.9)	666 (59.5)	
Total Dose, n (%)	5000–5999 cGy	0	332 (2.2)		332 (29.7)	n/a
	6000–6599 cGy		787 (5.3)		787 (70.3)	
	6600–6999 cGy		3328 (22.4)	3328 (24.2)		
	7000+ cGy		10422 (70.1)	10422 (75.8)		

Continuous variables are summarized as median (IQR), and categorical variables as frequencies with percentages. Percentages in the Overall column are calculated using the entire cohort as the denominator. Percentages in the DDRT and SDRT columns are calculated within each treatment group for the Total Dose row because dose categories define cohort membership. P-values reflect comparisons between SDRT and DDRT cohorts.

### Patient and treatment characteristics

Baseline demographic characteristics were similar between groups. Patients in the DDRT cohort were slightly older (median age 64 vs. 63 years, p < 0.001) with a modest overrepresentation of individuals aged 70 years or older. Sex and racial distributions did not significantly differ between treatment groups. The use of DDRT varied substantially by facility type: 50.3% of DDRT patients were treated at academic or NCI-designated cancer centers, compared with 40.0% of those receiving SDRT (p < 0.001). Geographic variation was also evident, with higher rates of DDRT in the South Atlantic regions and lower adoption in New England and the West South Central region (p < 0.001).

Chemotherapy use differed meaningfully between cohorts. SDRT patients were more commonly treated with single-agent first-course chemotherapy, while DDRT patients had higher rates of multi-agent chemotherapy and higher rates of no chemotherapy (p < 0.001). Immunotherapy use during the primary course of treatment, while uncommon overall, was more frequently observed in the DDRT group.

### Predictors of dose de-escalation

On multivariable logistic regression, treatment at an Academic/Research Program, compared with a Community Cancer Program (reference category) was independently associated with receipt of DDRT (Odds Ratio: 1.76, 95% Confidence Interval 1.31-2.43, p<0.001). Patients who were diagnosed more recently were also associated with higher likelihood of DDRT use, indicating a gradual rise in adoption over time. Geographic location demonstrated strong influence, with certain regions showing significantly higher or lower odds of de-escalation independent of patient and tumor factors. Multicollinearity analysis demonstrated adjusted generalized variance inflation factors (GVIF^(1/(2×Df))) ranging from 1.00 to 1.21 across all covariates included in the multivariable logistic regression model (**Supplementary Table 1**).

### Overall survival analysis

**Figure 2:**
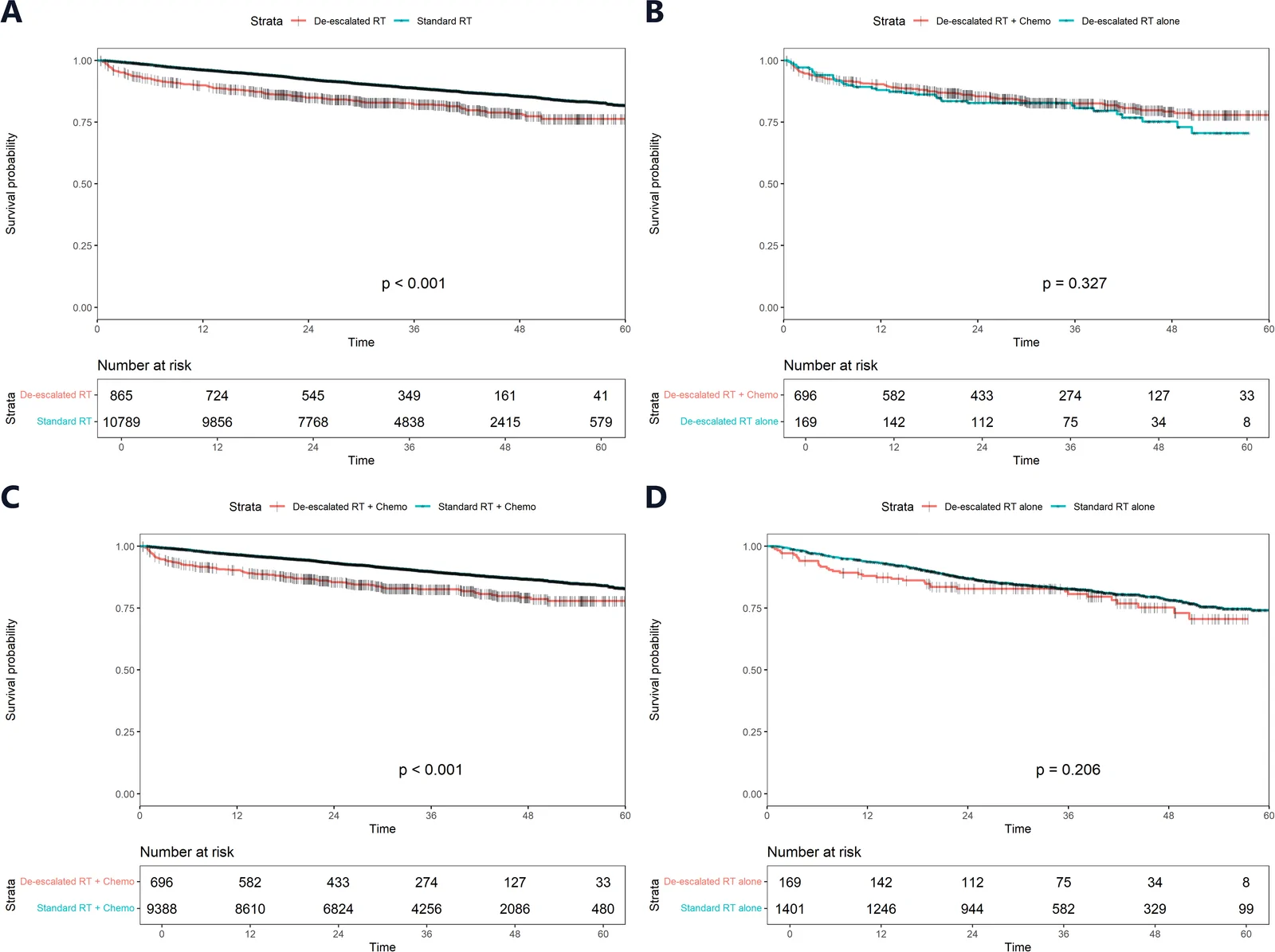
Kaplan–Meier curves of overall survival according to radiation dose and chemotherapy status with two-month landmark analysis. **(A)** Overall survival for patients receiving standard-dose radiation therapy, stratified by receipt of chemotherapy. **(B)** Overall survival for patients receiving dose-de-escalated radiation therapy, stratified by receipt of chemotherapy. **(C)** Overall survival among patients who received chemotherapy, stratified by radiation dose group (standard-dose vs. de-escalated). **(D)** Overall survival among patients who did not receive chemotherapy, stratified by radiation dose group (standard-dose vs. de-escalated).

Median follow-up was 38.2 months overall (38.2 months for SDRT and 37.4 months for DDRT), and median OS was not reached in either cohort. Three-year OS was 88.9% for the SDRT cohort compared with 82.1% for the DDRT cohort. Dose de-escalation was associated with inferior OS compared with standard-dose treatment (**Figures 2A–D**). Given the observed differences in chemotherapy use, survival analyses were stratified by receipt of chemotherapy. Among patients who received first-course chemotherapy, DDRT remained associated with inferior OS (three-year OS 82.5% vs. 89.8%, p < 0.001). In contrast, among patients who did not receive chemotherapy, OS did not significantly differ between groups (p = 0.206). However, this subgroup was small and likely underpowered to detect a meaningful difference.

On multivariable Cox proportional hazards regression (**Table 2**), receipt of DDRT remained independently associated with inferior OS compared with SDRT (HR 1.72, 95% CI 1.43-2.07, p<0.001). Older age (≥70 years; HR 1.67, 95% CI 1.45-1.92, p<0.001), increasing Charlson-Deyo comorbidity score, and lack of private insurance were also independently associated with worse survival. Treatment at an academic/research program was not independently associated with OS (HR 0.98, 95% CI 0.76-1.26, p=0.880). Geographic variation persisted, with treatment in the Pacific, South Atlantic, and West South Central regions associated with improved survival relative to the East North Central region. Higher neighborhood median income was associated with improved survival, whereas treatment delivered entirely at another facility was associated with modestly worse survival (HR 1.19, 95% CI 1.01-1.41, p=0.036).

**Table 2:** Multivariable logistic regression predicting for receipt of dose-de-escalated radiation therapy (DDRT) and multivariate cox regression predicting for overall survival.

Variable	Logistic regression results (OR, 95% CI, p value)	Cox regression results (HR, 95% CI, p value)
Age		
<70	OR=1, reference	HR=1, reference
>=70	OR: 1.17, 95% CI 0.98-1.40, p=0.079	HR: 1.67, 95% CI 1.45-1.92, p<0.001
Sex		
Female	OR=1, reference	HR=1, reference
Male	OR: 0.90, 95% CI 0.74-1.11, p=0.319	HR: 1.18, 95% CI 0.98-1.42, p=0.081
Race		
White	OR=1, reference	HR=1, reference
Asian	OR: 0.81, 95% CI 0.34-1.65, p=0.606	HR: 0.97, 95% CI 0.48-1.96, p=0.939
Black	OR: 0.83, 95% CI 0.58-1.16, p=0.303	HR: 0.89, 95% CI 0.67-1.19, p=0.439
Native American	OR: 0.92, 95% CI 0.15-3.13, p=0.913	HR: 0.56, 95% CI 0.08-4.01, p=0.565
Other	OR: 1.31, 95% CI 0.78-2.07, p=0.281	HR: 0.79, 95% CI 0.46-1.34, p=0.385
Year of diagnosis		
2018	OR=1, reference	HR=1, reference
2019	OR: 1.00, 95% CI 0.80-1.26, p=0.971	HR: 1.00, 95% CI 0.86-1.17, p=0.999
2020	OR: 1.00, 95% CI 0.80-1.26, p=0.976	HR: 0.86, 95% CI 0.72-1.03, p=0.093
2021	OR: 1.10, 95% CI 0.89-1.38, p=0.378	HR: 1.06, 95% CI 0.88-1.27, p=0.536
2022	OR: 1.06, 95% CI 0.85-1.33, p=0.591	n/a
Facility type		
Community Cancer Program	OR=1, reference	HR=1, reference
Academic/Research Program (includes NCI-designated comprehensive cancer centers)	OR: 1.76, 95% CI 1.31-2.43, p<0.001	HR: 0.98, 95% CI 0.76-1.26, p=0.880
Comprehensive Community Cancer Program	OR: 0.98, 95% CI 0.72-1.35, p=0.884	HR: 1.19, 95% CI 0.93-1.52, p=0.167
Integrated Network Cancer Program	OR: 1.11, 95% CI 0.80-1.56, p=0.552	HR: 1.20, 95% CI 0.92-1.56, p=0.179
Facility location		
East North Central (IL, IN, MI, OH, WI)	OR=1, reference	HR=1, reference
East South Central (AL, KY, MS, TN)	OR: 1.71, 95% CI 1.26-2.30, p<0.001	HR: 0.90, 95% CI 0.69-1.15, p=0.393
Middle Atlantic (NJ, NY, PA)	OR: 1.07, 95% CI 0.81-1.42, p=0.611	HR: 0.86, 95% CI 0.69-1.08, p=0.198
Mountain (AZ, CO, ID, MT, NM, NV, UT, WY)	OR: 1.53, 95% CI 1.08-2.14, p=0.015	HR: 0.85, 95% CI 0.64-1.13, p=0.266
New England (CT, MA, ME, NH, RI, VT)	OR: 0.43, 95% CI 0.27-0.67, p<0.001	HR: 0.87, 95% CI 0.66-1.14, p=0.306
Pacific (AK, CA, HI, OR, WA)	OR: 1.34, 95% CI 1.02-1.75, p=0.032	HR: 0.70, 95% CI 0.56-0.87, p=0.002
South Atlantic (DC, DE, FL, GA, MD, NC, SC, VA, WV)	OR: 1.87, 95% CI 1.51-2.33, p<0.001	HR: 0.79, 95% CI 0.66-0.94, p=0.008
West North Central (IA, KS, MN, MO, ND, NE, SD)	OR: 1.52, 95% CI 1.14-2.02, p=0.004	HR: 0.86, 95% CI 0.67-1.09, p=0.215
West South Central (AR, LA, OK, TX)	OR: 0.76, 95% CI 0.54-1.06, p=0.112	HR: 0.67, 95% CI 0.52-0.87, p=0.003
Urban/rural classification		
Metro: ≥1 million population	OR=1, reference	HR=1, reference
Metro: <250,000 population	OR: 0.98, 95% CI 0.76-1.26, p=0.880	HR: 0.85, 95% CI 0.69-1.05, p=0.138
Metro: 250,000-999,999 population	OR: 0.96, 95% CI 0.80-1.14, p=0.629	HR: 0.92, 95% CI 0.79-1.08, p=0.315
Rural: <2,500 population, adjacent to metro	OR: 1.03, 95% CI 0.53-1.83, p=0.917	HR: 0.72, 95% CI 0.41-1.29, p=0.275
Rural: <2,500 population, not adjacent to metro	OR: 0.97, 95% CI 0.47-1.80, p=0.934	HR: 0.69, 95% CI 0.36-1.30, p=0.247
Urban:≥20,000 population, adjacent to metro	OR: 1.14, 95% CI 0.82-1.56, p=0.422	HR: 0.94, 95% CI 0.72-1.23, p=0.665
Urban: ≥20,000 population, not adjacent to metro	OR: 0.55, 95% CI 0.23-1.12, p=0.137	HR: 0.95, 95% CI 0.58-1.56, p=0.839
Urban: 2,500-19,999 population, adjacent to metro	OR: 1.31, 95% CI 0.98-1.74, p=0.065	HR: 0.91, 95% CI 0.71-1.17, p=0.463
Urban: 2,500-19,999 population, not adjacent to metro	OR: 1.31, 95% CI 0.86-1.95, p=0.189	HR: 0.82, 95% CI 0.55-1.20, p=0.305
Insurance status		
Private Insurance/Managed Care	OR=1, reference	HR=1, reference
Insurance Status Unknown	OR: 0.87, 95% CI 0.36-1.76, p=0.719	HR: 2.44, 95% CI 1.42-4.18, p=0.001
Medicaid	OR: 1.07, 95% CI 0.78-1.43, p=0.675	HR: 1.71, 95% CI 1.33-2.21, p<0.001
Medicare	OR: 1.12, 95% CI 0.94-1.33, p=0.191	HR: 1.79, 95% CI 1.53-2.10, p<0.001
Not Insured	OR: 1.38, 95% CI 0.86-2.12, p=0.160	HR: 2.76, 95% CI 1.91-3.98, p<0.001
Other Government	OR: 0.89, 95% CI 0.61-1.27, p=0.545	HR: 1.75, 95% CI 1.30-2.36, p<0.001
Percent no high school degree quartiles from 2000		
< 5.0%	OR=1, reference	HR=1, reference
5.0% - 9.0%	OR: 0.88, 95% CI 0.73-1.06, p=0.182	HR: 1.16, 95% CI 0.98-1.39, p=0.092
9.1% - 15.2%	OR: 0.88, 95% CI 0.70-1.09, p=0.248	HR: 1.17, 95% CI 0.95-1.43, p=0.137
15.3%+	OR: 0.79, 95% CI 0.59-1.04, p=0.097	HR: 1.28, 95% CI 1.01-1.63, p=0.040
Median income quartile in 2020		
< $46,277	OR=1, reference	HR=1, reference
$46,277 - $57,856	OR: 0.86, 95% CI 0.67-1.10, p=0.229	HR: 0.99, 95% CI 0.82-1.21, p=0.949
$57,857 - $74,062	OR: 0.87, 95% CI 0.67-1.13, p=0.281	HR: 0.77, 95% CI 0.62-0.96, p=0.020
$74,063+	OR: 0.91, 95% CI 0.68-1.21, p=0.502	HR: 0.76, 95% CI 0.60-0.97, p=0.028
Best Charlson Deyo score		
0	OR=1, reference	HR=1, reference
1	OR: 1.05, 95% CI 0.86-1.27, p=0.651	HR: 1.31, 95% CI 1.12-1.53, p=0.001
2	OR: 1.31, 95% CI 0.94-1.78, p=0.101	HR: 1.34, 95% CI 1.04-1.74, p=0.023
3 or more	OR: 0.73, 95% CI 0.47-1.08, p=0.133	HR: 2.36, 95% CI 1.89-2.94, p<0.001
Chemotherapy treatment status		
Single-agent chemotherapy administered as first course therapy	OR=1, reference	HR=1, reference
Chemotherapy administered as first course therapy	OR: 1.44, 95% CI 0.93-2.15, p=0.085	HR: 1.67, 95% CI 1.15-2.42, p=0.007
Contraindicated due to patient risk factors	OR: 1.33, 95% CI 0.61-2.55, p=0.429	HR: 2.02, 95% CI 1.37-2.98, p<0.001
Multiagent chemotherapy administered as first course therapy	OR: 1.48, 95% CI 1.21-1.81, p<0.001	HR: 1.37, 95% CI 1.15-1.64, p<0.001
None	OR: 1.86, 95% CI 1.55-2.22, p<0.001	HR: 1.56, 95% CI 1.34-1.82, p<0.001
Not administered; patient/family refused	OR: 1.19, 95% CI 0.55-2.27, p=0.627	HR: 2.19, 95% CI 1.38-3.47, p=0.001
Not administered; physician recommended, but not given	OR: 0.00, 95% CI nan-96.46, p=0.943	HR: 0.00, 95% CI 0.00-inf, p=0.984
Radiation location		
All RT at this facility	OR=1, reference	HR=1, reference
All RT elsewhere	OR: 1.19, 95% CI 0.98-1.44, p=0.072	HR: 1.19, 95% CI 1.01-1.41, p=0.036
Boost radiation at this facility, regional elsewhere	OR: 1.12, 95% CI 0.06-6.34, p=0.917	HR: 1.36, 95% CI 0.19-9.73, p=0.762
Other/Unknown	OR: 0.46, 95% CI 0.16-1.04, p=0.096	HR: 1.00, 95% CI 0.50-2.02, p=0.996
Regional treatment at this facility, boost elsewhere	OR: 3.94, 95% CI 1.27-10.31, p=0.009	HR: 1.83, 95% CI 0.59-5.73, p=0.298
Dose de escalation		
SDRT	n/a	HR=1, reference
DDRT	n/a	HR: 1.72, 95% CI 1.43-2.07, p<0.001

A sensitivity analysis restricted to patients receiving DDRT compared outcomes between those treated with 50.0-59.9 Gy and 60.0-65.9 Gy. On multivariable Cox regression, receipt of 60.0-65.9 Gy was independently associated with improved OS compared with 50.0-59.9 Gy (HR 0.41, 95% CI 0.28-0.60, p<0.001). Multivariable logistic and Cox regression results for this analysis are provided in **Supplementary Table 2**.

### Outcomes by facility type among patients receiving DDRT

**Figure 3:**
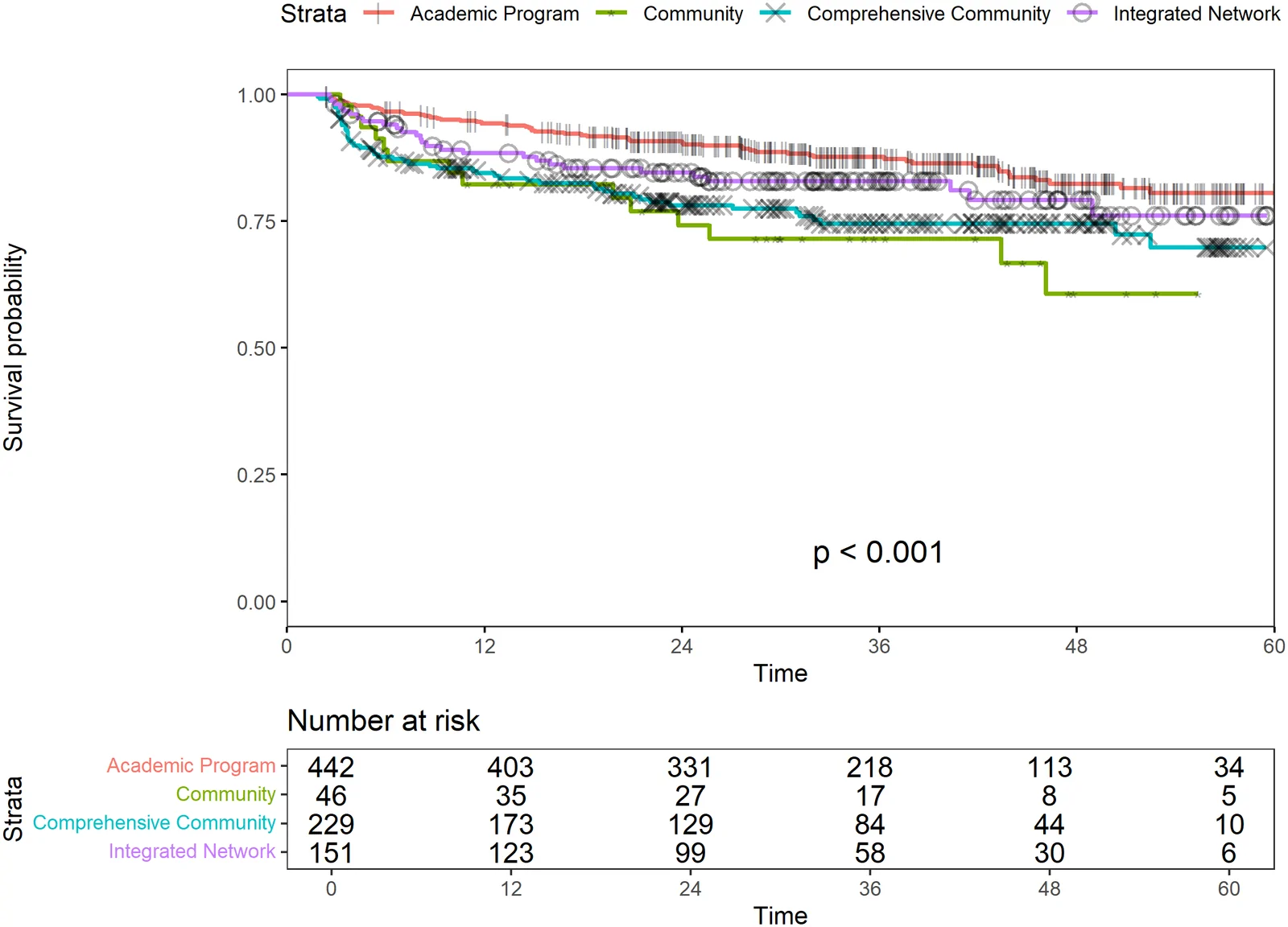
Kaplan–Meier overall survival among patients receiving dose-de-escalated radiation therapy stratified by treatment at academic versus non-academic centers.

Among patients who received dose-de-escalated radiation therapy, OS differed according to treatment facility type. Among patients who received DDRT, patients treated at academic centers had superior OS compared with those treated at non-academic centers (log-rank p < 0.001) (**Figure 3**). This finding suggests that outcomes among patients receiving DDRT vary by treatment setting.

## Discussion

In this large, contemporary NCDB analysis, we found that radiation dose de-escalation for p16-positive OPSCC was uncommon overall, occurring in only 7.5% of patients between 2018 and 2022. Adoption was most frequent at academic centers and in specific geographic regions, underscoring variability in practice. Critically, patients treated with de-escalated RT had significantly inferior OS compared with those who received standard, mirroring the negative results of NRG-HN005 (15).

The early enthusiasm for radiation dose reduction in HPV-positive OPSCC stemmed largely from single-institution and phase II studies (8, 9, 11, 13, 14, 24). Trials such as MSKCC 30 ROC, ECOG 3311, and ECOG 1308 demonstrated early promising locoregional control with 30–60 Gy regimens in carefully selected low-risk patients, typically never- or light-smokers with early T stage and favorable performance status, in either the definitive or the postoperative setting (12, 14, 25). These findings suggested that de-intensification could reduce long-term toxicities while maintaining disease control, spurring subsequent randomized investigations. However, a phase III update of the Mayo Clinic’s post-operative de-escalation study especially in pN2 disease was inferior to the standard postoperative dose (26). In addition, NRG-HN005 failed to confirm non-inferiority of de-escalated arms in the definitive setting, emphasizing that even within low-risk populations, standard-dose chemoradiation remains the safest benchmark (15).

Our study provides real-world evidence that de-escalation has nonetheless diffused into practice, particularly at academic centers. Although several prospective studies have evaluated de-escalation strategies, including reductions in elective nodal volumes or radiation fields, the present analysis is limited to de-escalation through reduction in total prescribed radiation dose. With over 1100 low-risk HPV-positive OPSCC DDRT patients identified in the NCDB and the four largest radiation dose de-escalation trials (9–11, 15) comprising less than 400 of these patients, it is clear that the majority of DDRT patients were treated off protocol. Academic centers are often more comfortable adopting investigational approaches, guided by preliminary evidence and patient demand for less toxic therapy. By contrast, community programs may adhere more strictly to guideline-recommended dosing and are less likely to treat per (but off) protocol (16, 17). While innovation is essential, our data suggest that extrapolation of de-escalation outside trial protocols has been associated with worse survival at the population level. Interestingly, Charlson-Deyo comorbidity score was independently associated with worse OS but was not associated with receipt of DDRT. This finding suggests that dose de-escalation was not preferentially used in patients with greater measured comorbidity, although selection based on unmeasured factors, such as performance status or physician judgment, cannot be excluded.

Geographic differences further highlight disparities in access to trials, referral patterns, and provider practice culture. Higher rates of de-escalation in regions such as the South Atlantic and Pacific may reflect concentrations of academic programs or early trial activity, whereas lower adoption in New England and West South Central states may correspond to more conservative practice norms.

Although patients receiving DDRT at academic centers demonstrated superior unadjusted OS compared with those treated at non-academic centers, treatment at an academic facility was not independently associated with OS after adjustment for demographic, socioeconomic, clinical, and treatment-related factors. This finding suggests that the apparent survival advantage observed with DDRT at academic versus non-academic centers on univariable analysis is likely attributable to differences in patient selection and case mix rather than facility type itself. Receipt of DDRT remained independently associated with worse OS in the multivariable Cox model, reinforcing the robustness of the observed association between radiation dose de-escalation and inferior survival despite adjustment for measured confounders. Among patients who received first-course chemotherapy, dose de-escalation remained associated with inferior OS versus standard-dose radiation therapy. This suggests that when DDRT is used in the setting where full-intensity chemoradiation is otherwise feasible, reduced radiation dose may compromise disease control and that chemotherapy does not appear to compensate for decreased radiation doses. In contrast, among patients who did not receive chemotherapy, OS was similar between DDRT and SDRT groups. However, patients who do not receive chemotherapy in definitive treatment are typically clinically distinct, often with greater comorbidities or treatment contraindications, and therefore may not represent intentional de-escalation candidates. Taken together, these stratified findings indicate that the survival disadvantage associated with DDRT is most evident in the population typically considered appropriate for curative-intent chemoradiation, while similarity in outcomes among non-chemotherapy patients likely reflects different patient selection dynamics rather than equivalence of de-escalated radiotherapy. Within the DDRT cohort, patients treated with 60.0-65.9 Gy demonstrated improved OS compared with those treated with 50.0-59.9 Gy, suggesting that outcomes may differ across de-escalated dose regimens rather than representing a uniform effect of all doses below 66 Gy.

The association between de-escalation and inferior survival warrants cautious interpretation. Although multivariable analysis adjusted for measured confounders, the NCDB lacks granular data on smoking history, HPV positivity, HPV subtype, RT field design, and compliance, factors central to patient selection in early-phase studies (15). It is plausible that clinicians at academic centers preferentially chose de-escalation for patients they perceived as low risk, but residual confounding or selection bias cannot be excluded. Although patients receiving DDRT were slightly older, age was included in the multivariable Cox model and DDRT remained an independent predictor of OS. Nevertheless, as with any retrospective observational study, residual confounding from unmeasured factors cannot be excluded. Nevertheless, the survival disadvantage observed in this population-level dataset parallels the cautionary signal from randomized evidence, reinforcing that dose reduction should remain investigational.

Limitations of our study include the retrospective nature of the NCDB, absence of recurrence and toxicity data, and inability to assess functional outcomes, which are a primary rationale for de-escalation. Additionally, the NCDB does not reliably capture the number of fractions delivered per week, precluding assessment of altered or accelerated fractionation regimens. Consequently, treatment classification was based solely on total nominal radiation dose. Because fractionation schedules (e.g., accelerated or hyperfractionated courses delivering equivalent or higher biological effective doses) cannot be confirmed, it cannot be stated with certainty that a total dose of 66 Gy universally represents an intended dose de-escalation rather than an altered fractionation schedule or standard variant. This potential misclassification of biologically effective dose remains an inherent limitation of registry-based dose grouping. Furthermore, annual head and neck cancer treatment volume at individual facilities was not available in the dataset used for this analysis and therefore could not be incorporated into the multivariable models. Facility volume may be associated with both treatment selection and outcomes and represents a potential source of residual confounding. Several categories for radiation location contained very small numbers of patients, resulting in unstable estimates and wide confidence intervals. Accordingly, findings for these categories should be interpreted with caution. Finally, an additional limitation is that the NCDB does not provide data about the clinical rationale underlying treatment selection and does not capture performance status or treatment-related toxicity, limiting our ability to compare the baselines fitness of DDRT versus standard dose patients and to determine whether reduced radiation dose reflected planned de-escalation or incomplete treatment. Despite these constraints, our analysis represents one of the largest national assessments of dose de-intensification patterns in HPV-positive OPSCC, providing crucial insight into how clinical trial concepts translate into practice.

## Conclusion

In conclusion, radiation dose de-escalation remains infrequent in the United States but is disproportionately adopted at academic centers and in select regions, likely reflecting trial participation and patient selection strategies. Patients who underwent de-escalated RT experienced inferior OS, consistent with NRG-HN005. These findings emphasize that until robust biomarkers or prospective evidence identify patients who can safely undergo reduced-dose RT, standard-dose chemoradiation should remain the treatment of choice outside clinical trials (27–29).

## Data Availability

The datasets presented in this article are not readily available because the data are available only to approved investigators through the NCDB Participant User File (PUF) program under a data use agreement. Requests to access the datasets should be directed to ncdb_puf@facs.org.
