# Supplementary material for: Do not try this at home: patterns and outcomes of radiation dose de-escalation in p16-positive oropharyngeal carcinoma
Source: Front Oncol. 2026 Sep 7;16:1929183. doi: 10.3389/fonc.2026.1929183 (PMC13590389; doi:10.3389/fonc.2026.1929183)
Supplement: Supplementary file 1 [file SupplementaryFile1.docx]

**Supplementary Table 1** Generalized variance inflation factor (GVIF) analysis for covariates included in the multivariable logistic regression model.

| **Variable** | **Adjusted GVIF** |
| --- | --- |
| Age at least 70 | 1.21 |
| Sex | 1.01 |
| Race | 1.01 |
| Year of Diagnosis | 1.00 |
| Facility Type | 1.03 |
| Facility Location | 1.03 |
| Urban/Rural Classification | 1.02 |
| Insurance Status | 1.05 |
| Percent No High School Degree Quartiles | 1.13 |
| Median Income Quartile in 2020 | 1.16 |
| Best Charlson Deyo Score | 1.01 |
| Radiation Location | 1.01 |
| Chemotherapy Treatment Status | 1.01 |
