# Supplementary material for: Do not try this at home: patterns and outcomes of radiation dose de-escalation in p16-positive oropharyngeal carcinoma
Source: Front Oncol. 2026 Sep 7;16:1929183. doi: 10.3389/fonc.2026.1929183 (PMC13590389; doi:10.3389/fonc.2026.1929183)
Supplement: Supplementary file 2 [file SupplementaryFile2.docx]

Supplementary Table 2 Multivariable logistic regression analysis predicting factors associated with receipt of 6000 to 6599 cGy versus 5000 to 5999 cGy among patients receiving dose de-escalated radiation therapy (DDRT) and multivariable Cox regression analysis predicting overall survival.

| **Variable** | **Logistic Regression Results (OR, 95% CI, p value)** | **Cox Regression Results (HR, 95% CI, p value)** |
| --- | --- | --- |
| Age |  |  |
| <70 | OR=1, reference | HR=1, reference |
| >=70 | OR: 0.76, 95% CI 0.50-1.15, p=0.191 | HR: 2.00, 95% CI 1.28-3.13, p=0.002 |
| Sex |  |  |
| Female | OR=1, reference | HR=1, reference |
| Male | OR: 1.33, 95% CI 0.83-2.12, p=0.228 | HR: 0.91, 95% CI 0.56-1.49, p=0.707 |
| Race |  |  |
| White | OR=1, reference | HR=1, reference |
| Asian | OR: 2765413.62, 95% CI 0.00-nan, p=0.975 | HR: 0.00, 95% CI 0.00-inf, p=0.996 |
| Black | OR: 0.96, 95% CI 0.43-2.28, p=0.928 | HR: 0.32, 95% CI 0.09-1.16, p=0.082 |
| Native American | OR: 0.15, 95% CI 0.01-4.19, p=0.204 | HR: 0.00, 95% CI 0.00-inf, p=0.998 |
| Other | OR: 1.72, 95% CI 0.56-6.53, p=0.374 | HR: 0.97, 95% CI 0.28-3.41, p=0.964 |
| Year of Diagnosis |  |  |
| 2018 | OR=1, reference | HR=1, reference |
| 2019 | OR: 1.41, 95% CI 0.83-2.41, p=0.206 | HR: 0.93, 95% CI 0.56-1.55, p=0.773 |
| 2020 | OR: 1.33, 95% CI 0.78-2.29, p=0.296 | HR: 0.90, 95% CI 0.52-1.56, p=0.709 |
| 2021 | OR: 1.22, 95% CI 0.73-2.05, p=0.443 | HR: 1.31, 95% CI 0.76-2.26, p=0.335 |
| 2022 | OR: 1.71, 95% CI 1.00-2.91, p=0.048 | N/a |
| Facility Type |  |  |
| Community Cancer Program | OR=1, reference | HR=1, reference |
| Academic/Research Program (includes NCI-designated comprehensive cancer centers) | OR: 1.45, 95% CI 0.71-2.90, p=0.300 | HR: 0.51, 95% CI 0.24-1.07, p=0.076 |
| Comprehensive Community Cancer Program | OR: 0.82, 95% CI 0.40-1.66, p=0.595 | HR: 0.83, 95% CI 0.41-1.71, p=0.619 |
| Integrated Network Cancer Program | OR: 1.59, 95% CI 0.73-3.41, p=0.240 | HR: 0.61, 95% CI 0.28-1.34, p=0.221 |
| Facility Location |  |  |
| East North Central (IL, IN, MI, OH, WI) | OR=1, reference | HR=1, reference |
| East South Central (AL, KY, MS, TN) | OR: 4.32, 95% CI 2.22-8.64, p<0.000 | HR: 1.33, 95% CI 0.68-2.61, p=0.403 |
| Middle Atlantic (NJ, NY, PA) | OR: 1.41, 95% CI 0.78-2.58, p=0.257 | HR: 0.82, 95% CI 0.39-1.72, p=0.609 |
| Mountain (AZ, CO, ID, MT, NM, NV, UT, WY) | OR: 3.43, 95% CI 1.54-8.07, p=0.003 | HR: 0.62, 95% CI 0.22-1.78, p=0.378 |
| New England (CT, MA, ME, NH, RI, VT) | OR: 1.36, 95% CI 0.52-3.69, p=0.534 | HR: 0.72, 95% CI 0.20-2.61, p=0.621 |
| Pacific (AK, CA, HI, OR, WA) | OR: 3.48, 95% CI 1.89-6.52, p<0.001 | HR: 0.55, 95% CI 0.26-1.15, p=0.112 |
| South Atlantic (DC, DE, FL, GA, MD, NC, SC, VA, WV) | OR: 4.56, 95% CI 2.73-7.67, p<0.001 | HR: 0.93, 95% CI 0.53-1.65, p=0.817 |
| West North Central (IA, KS, MN, MO, ND, NE, SD) | OR: 2.96, 95% CI 1.50-5.95, p=0.002 | HR: 0.70, 95% CI 0.29-1.72, p=0.439 |
| West South Central (AR, LA, OK, TX) | OR: 4.52, 95% CI 2.01-10.91, p<0.001 | HR: 0.27, 95% CI 0.08-0.95, p=0.042 |
| Urban/Rural Classification |  |  |
| Metro: ?1 million population | OR=1, reference | HR=1, reference |
| Metro: <250,000 population | OR: 0.68, 95% CI 0.38-1.25, p=0.207 | HR: 0.74, 95% CI 0.36-1.52, p=0.410 |
| Metro: 250,000-999,999 population | OR: 0.81, 95% CI 0.52-1.26, p=0.341 | HR: 0.88, 95% CI 0.53-1.45, p=0.612 |
| Rural: <2,500 population, adjacent to metro | OR: 0.69, 95% CI 0.18-3.04, p=0.597 | HR: 0.81, 95% CI 0.11-6.19, p=0.838 |
| Rural: <2,500 population, not adjacent to metro | OR: 0.52, 95% CI 0.12-2.40, p=0.378 | HR: 1.69, 95% CI 0.37-7.70, p=0.499 |
| Urban:?20,000 population, adjacent to metro | OR: 1.17, 95% CI 0.54-2.62, p=0.699 | HR: 0.63, 95% CI 0.25-1.57, p=0.325 |
| Urban: ?20,000 population, not adjacent to metro | OR: 0.40, 95% CI 0.08-3.00, p=0.300 | HR: 0.00, 95% CI 0.00-inf, p=0.996 |
| Urban: 2,500-19,999 population, adjacent to metro | OR: 1.32, 95% CI 0.66-2.78, p=0.453 | HR: 0.28, 95% CI 0.09-0.80, p=0.018 |
| Urban: 2,500-19,999 population, not adjacent to metro | OR: 1.09, 95% CI 0.42-3.01, p=0.869 | HR: 1.23, 95% CI 0.43-3.47, p=0.700 |
| Insurance Status |  |  |
| Private Insurance / Managed Care | OR=1, reference | HR=1, reference |
| Insurance Status Unknown | OR: 0.44, 95% CI 0.08-2.51, p=0.320 | HR: 5.19, 95% CI 1.08-24.86, p=0.039 |
| Medicaid | OR: 0.74, 95% CI 0.38-1.50, p=0.393 | HR: 3.52, 95% CI 1.69-7.34, p=0.001 |
| Medicare | OR: 0.64, 95% CI 0.42-0.96, p=0.030 | HR: 1.70, 95% CI 0.99-2.93, p=0.055 |
| Not Insured | OR: 0.32, 95% CI 0.12-0.89, p=0.022 | HR: 3.25, 95% CI 1.04-10.17, p=0.043 |
| Other Government | OR: 1.80, 95% CI 0.67-5.85, p=0.280 | HR: 2.22, 95% CI 0.79-6.24, p=0.132 |
| Percent No High School Degree Quartiles from 2000 |  |  |
| < 5.0% | OR=1, reference | HR=1, reference |
| 5.0% - 9.0% | OR: 0.82, 95% CI 0.52-1.30, p=0.400 | HR: 1.41, 95% CI 0.82-2.44, p=0.218 |
| 9.1% - 15.2% | OR: 0.53, 95% CI 0.31-0.89, p=0.016 | HR: 1.57, 95% CI 0.85-2.88, p=0.146 |
| 15.3%+ | OR: 0.54, 95% CI 0.29-1.02, p=0.057 | HR: 1.83, 95% CI 0.89-3.76, p=0.101 |
| Median Income Quartile in 2020 |  |  |
| < $46,277 | OR=1, reference | HR=1, reference |
| $46,277 - $57,856 | OR: 1.27, 95% CI 0.70-2.28, p=0.430 | HR: 1.96, 95% CI 0.98-3.89, p=0.056 |
| $57,857 - $74,062 | OR: 0.87, 95% CI 0.47-1.60, p=0.662 | HR: 1.38, 95% CI 0.66-2.90, p=0.398 |
| $74,063+ | OR: 0.74, 95% CI 0.38-1.43, p=0.376 | HR: 1.63, 95% CI 0.75-3.57, p=0.218 |
| Best Charlson Deyo Score |  |  |
| 0 | OR=1, reference | HR=1, reference |
| 1 | OR: 0.92, 95% CI 0.59-1.46, p=0.724 | HR: 1.41, 95% CI 0.89-2.22, p=0.142 |
| 2 | OR: 0.91, 95% CI 0.45-1.93, p=0.793 | HR: 1.47, 95% CI 0.77-2.83, p=0.246 |
| 3 or more | OR: 0.50, 95% CI 0.20-1.28, p=0.139 | HR: 2.17, 95% CI 0.93-5.07, p=0.072 |
| Chemotherapy Treatment Status |  |  |
| Single-agent chemotherapy administered as first course therapy | OR=1, reference | HR=1, reference |
| Chemotherapy administered as first course therapy | OR: 1.00, 95% CI 0.38-2.98, p=0.995 | HR: 1.14, 95% CI 0.26-5.04, p=0.861 |
| Contraindicated due to patient risk factors | OR: 0.44, 95% CI 0.09-2.06, p=0.301 | HR: 2.11, 95% CI 0.76-5.83, p=0.152 |
| Multiagent chemotherapy administered as first course therapy | OR: 0.38, 95% CI 0.24-0.60, p<0.001 | HR: 1.08, 95% CI 0.62-1.89, p=0.779 |
| None | OR: 1.30, 95% CI 0.84-2.04, p=0.240 | HR: 1.21, 95% CI 0.76-1.91, p=0.419 |
| Not administered; patient/family refused | OR: 1.08, 95% CI 0.24-5.83, p=0.918 | HR: 1.65, 95% CI 0.45-6.07, p=0.454 |
| Radiation Location |  |  |
| All RT at this facility | OR=1, reference | HR=1, reference |
| All RT elsewhere | OR: 0.69, 95% CI 0.45-1.06, p=0.086 | HR: 0.94, 95% CI 0.57-1.57, p=0.816 |
| Boost radiation at this facility, regional elsewhere | OR: 943703.96, 95% CI 0.00-nan, p=0.992 | HR: 0.00, 95% CI 0.00-inf, p=0.999 |
| Other/Unknown | OR: 0.58, 95% CI 0.08-5.72, p=0.612 | HR: 0.00, 95% CI 0.00-inf, p=0.997 |
| Regional treatment at this facility, boost elsewhere | OR: 0.37, 95% CI 0.04-2.82, p=0.350 | HR: 1.25, 95% CI 0.15-10.22, p=0.832 |
| Dose De Escalation Category |  |  |
| 5000-5999 cGy | N/a | HR=1, reference |
| 6000-6599 cGy | N/a | HR: 0.41, 95% CI 0.28-0.60, p<0.001 |
